# A natural loss-of-function deletion of the cytohesin 1 (Cyth1) gene in BALB/cByJ mice does not impact cardiomyocyte polyploidy

**DOI:** 10.1038/s41598-024-63667-8

**Published:** 2024-06-10

**Authors:** Ruolan Song, Hirofumi Watanabe, Kelsey Tjen, Baylee C. Westbury, Takako Makita, Ge Tao, Henry M. Sucov

**Affiliations:** 1 Department of Regenerative Medicine and Cell Biology, Medical University of South Carolina, Charleston, USA; 2 Department of Pediatrics, Medical University of South Carolina, Charleston, USA; 3 Present Address: Department of Pediatrics and Child Health, Nihon University School of Medicine, Tokyo, Japan

**Keywords:** Cytohesin, Polyploidy, BALB/cBy, Cell biology, Genetics

## Abstract

Mammalian cardiomyocytes (CMs) mostly become polyploid shortly after birth. Because this feature may relate to several aspects of heart biology, including regeneration after injury, the mechanisms that cause polyploidy are of interest. BALB/cJ and BALB/cByJ mice are highly related sister strains that diverge substantially in CM ploidy. We identified a large deletion in the Cyth1 gene that arose uniquely in BALB/cByJ mice that creates a null allele. The deletion also results in ectopic transcription of the downstream gene Dnah17, although this transcript is unlikely to encode a protein. By evaluating the natural null allele from BALB/cByJ and an engineered knockout allele in the C57BL/6J background, we determined that absence of Cyth1 does not by itself influence CM ploidy. The ready availability of BALB/cByJ mice may be helpful to other investigations of Cyth1 in other biological processes.

## Introduction

Natural phenotypic variation between inbred mouse strains has proven to be a powerful resource to identify new genes that are relevant to processes or traits of interest. In our past work^1^, we used this forward genetics strategy to map loci and identify new genes that are involved in cardiomyocyte (CM) polyploidy. Most cells of the body are diploid, containing two copies of the genome in one nucleus (notated as 1 × 2N; N is the haploid number of chromosomes). Polyploidy is a condition in which a single cell has additional duplicated copies of the full genome. In mammalian CMs and some other cell types (e.g., hepatocytes), polyploidy arises when diploid cells initiate cell cycle entry and S-phase DNA replication but followed by cell cycle interruption. This results in cells with one nucleus containing a tetraploid level of chromosomes (notated as 1 × 4N), or in cells containing two nuclei each with a 2N chromosome level (notated as 2 × 2N), depending on whether cell cycle interruption occurred before karyokinesis or before cytokinesis, respectively. Higher numbers of genomes, of nuclei, or of both can occur with subsequent cell cycle activity followed again by cell cycle interruption. This process is distinguished from polyploidy that results from cell fusion, as in skeletal muscle or syncytial trophoblasts, and is also distinguished from single chromosome aneuploidies, such as commonly occur in cancer cells. In the adult mouse (and other commonly studied mammals, including humans), typically > 90% of ventricular CMs are polyploid, and thus only a small percent are diploid.

While the phenomenon of CM polyploidy is of interest in its own right, it has added significance for its possible influence on CM proliferation and regeneration^2^. Polyploidy and proliferation are fundamentally related, as both start with entry into cell cycle and DNA replication in S-phase, followed by either cell cycle interruption (polyploidy) or completion (proliferation). In mice (and where studied, also most other mammalian species), essentially all embryonic CMs are diploid, and essentially all fetal CMs that enter cell cycle complete it. In the early neonatal heart, before the onset of polyploidy, injury causes reactivation of CM cell cycle activity which culminates in proliferation to fully regenerate the proper number of cells^3^. In normal mice midway through the first postnatal week, there is natural induction of cell cycle activity that is mostly followed by interruption, such that most CMs become polyploid at this time^4^. Concurrently, the heart’s ability to support CM proliferation is mostly lost^3,5^, and thereafter the heart responds to injury by scarring rather than by regeneration. Thus, CM polyploidy may be causative to loss of regenerative ability rather than only correlated temporally with it.

The specific mechanisms that cause CMs to interrupt or complete cell cycle are mostly unknown. We showed in our past work that the diploid CMs that are present in the normal adult heart are diploid because they mostly completed cell cycle in the neonatal period, instead of persisting from fetal life without cell cycle activity^6^. By surveying a large number of inbred mouse strains we showed that the percentage of diploid CMs in the adult mouse ventricle is a variable trait that is influenced by natural genetic polymorphisms^1^. These variants implicate the involvement of their corresponding genes in the interruption or completion of CM cell cycle.

The inbred mouse strains BALB/cJ and BALB/cByJ are considered as sister strains: they originated from an already-inbred BALB stock from which the two lines diverged in 1935 and were thereafter kept separate^7^. Several explanations could account for any current genetic and phenotypic differences between the two: a spontaneous mutation that arose after 1935 in one lineage, persistence in only one line of residual heterozygosity present in the common founder stock, or human error that introduced variation from another mouse background into one of the BALB lineages after 1935. Regardless of initial cause, the variants then became fixed to homozygosity in the current lines by inbreeding.

In our prior analysis, we demonstrated that BALB/cJ and BALB/cByJ mice have a striking phenotypic divergence in their CM ploidy profiles^1,8^. BALB/cJ mice are like most other mouse strains in having a low percentage of mononuclear CMs (6%) but with a high degree of nuclear diploidy within these (65%). In contrast, BALB/cByJ mice have a much higher mononuclear level (14%) but fewer of these nuclei are diploid (35%). By crossing these strains together and evaluating F1 mice, our data indicated that these trait differences could be explained by two (or more) loci, one X-linked (and likely not CM-autonomous) and one autosomal, in which both were recessive in BALB/cByJ relative to BALB/cJ^8^. Specifically, the X-linked recessive locus from BALB/cByJ resulted in higher mononuclear level, whereas the dominant autosomal locus from BALB/cJ resulted in a higher percentage of 2N nuclei within mononuclear CMs. These traits were separable and independent, such that the right direction of cross resulted in F1 mice with CMs that were 14% mononuclear with 65% nuclear diploidy^8^.

The alleles that confer these trait differences represent genes that are influential in karyokinesis or cytokinesis, at minimum in CMs if not also in other cell types. In this study, we identified a spontaneous loss of function allele of the autosomal Cyth1 gene in BALB/cByJ and evaluated this allele for its impact on CM polyploidy.

## Results

### Reciprocal expression of Cyth1 and Dnah17 in BALB/cJ and BALB/cByJ

We undertook an RNA-Seq analysis to investigate gene expression differences between BALB/cJ and BALB/cByJ in the neonatal heart. Ventricular tissue was isolated from postnatal day P4 neonates, which corresponds to the peak time when neonatal cardiomyocytes are in S-phase of the cell cycle and therefore will become polyploid or remain diploid. RNA was prepared from 4–5 hearts per sample in order to reach sufficient mass. Following assessment of RNA quality, 4 samples from BALB/cJ and 3 samples from BALB/cByJ were submitted for RNA-Seq evaluation. Differential gene expression differences are shown in Suppl. Table S1. A positive internal control for this analysis was expression of Zhx2, which uniquely in BALB/cJ carries an intronic retrovirus insertion that interferes with splicing and produces a polyadenylated fusion transcript lacking most of the mature Zhx2 mRNA sequence^9,10^. In our samples, Zhx2 was 18-fold higher in expression in BALB/cByJ compared to BALB/cJ. Because the alleles that influence CM ploidy are dominant in BALB/cJ, a loss-of-function mutation that is present only in this substrain, such as with Zhx2, is unlikely to explain strain phenotype differences.

**Figure 1:**
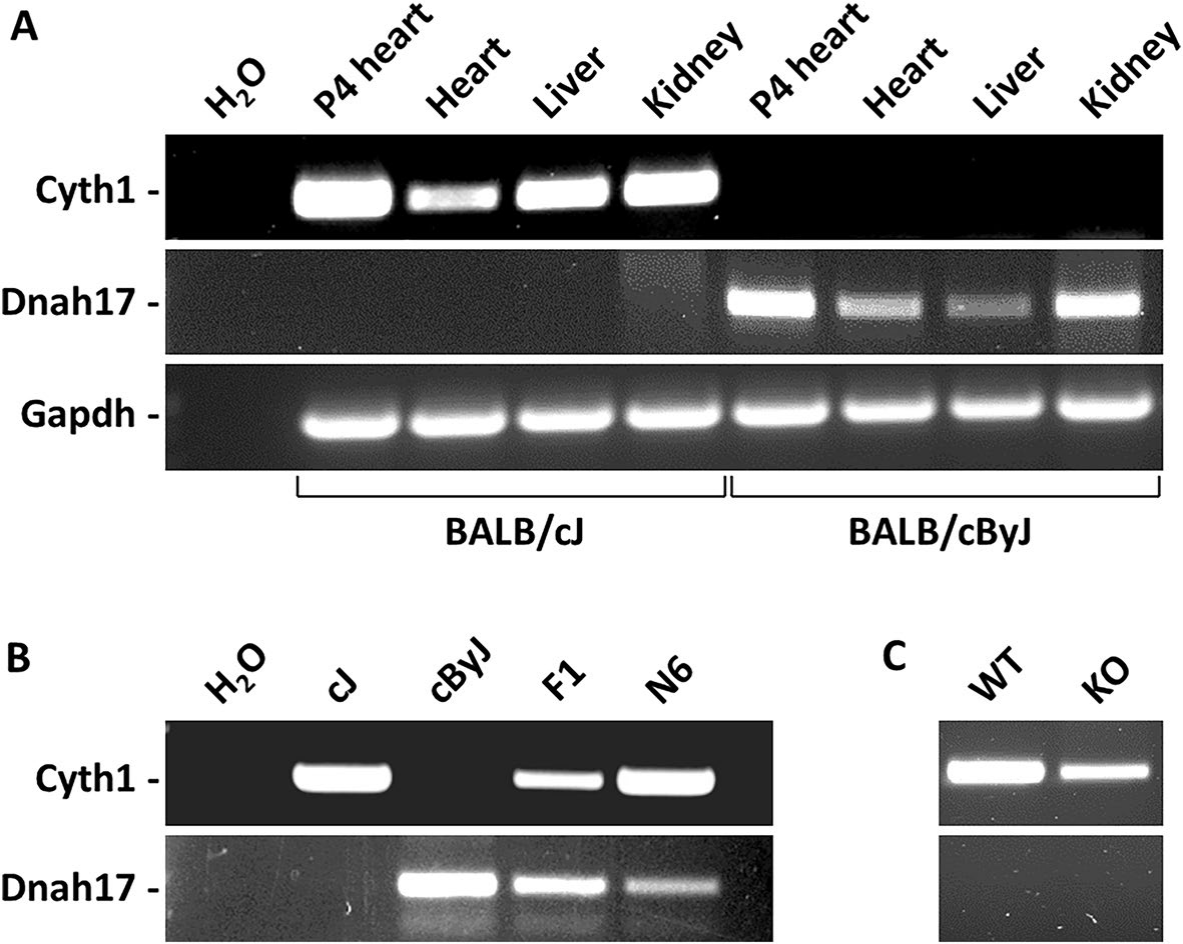
Divergent expression of Cyth1 and Dnah17 in BALB substrains evaluated by RT-PCR. (**A**) Expression in postnatal day 4 ventricle (P4 heart) and in adult tissues of BALB/cJ and BALB/cByJ mice. H_2_O is a no DNA input lane. (**B**) Expression profile in both parental strains, in F1 mice, and in N6 (6 generations backcrossed to BALB/cJ with selection for Cyth1 heterozygosity) mice. All samples are of ventricular tissue. (**C**) Expression in adult mice on an inbred C57BL/6 J background that are wild-type (WT) or homozygous for the engineered Cyth1 gene knockout (KO). The KO gene still expresses a stable transcript but which is nonfunctional (see text). Original uncropped gel images are shown in Supplementary Fig. S1.

The top differentially expressed gene in our comparison was cytohesin-1 (Cyth1), which was 180-fold higher in expression in BALB/cJ compared to BALB/cByJ. Another gene with substantial differential expression but in the opposite direction was dynein heavy chain 17 (Dnah17), which was 55-fold higher in expression in BALB/cByJ than in BALB/cJ. To confirm these results, we extracted RNA from neonatal ventricle and prepared cDNA, and amplified the Cyth1 and Dnah17 transcripts from both parental strains. As shown in Fig. 1A,B, we observed Cyth1 expression but no Dnah17 expression in BALB/cJ neonatal heart, and the opposite expression pattern for both genes in BALB/cByJ. This confirmed the outcome from the RNA-Seq analysis. Samples from neonatal heart, adult heart, and adult liver and kidney all showed the same opposite expression pattern for these two genes in the two BALB substrains (Fig. 1A).

In the ENCODE profile of gene expression across multiple tissues of C57BL/6 mice, Cyth1 is broadly expressed whereas Dnah17 is normally expressed only in the testes^11^. In datasets of gene expression in neonatal heart from several different mouse inbred strains, including C57BL/6 J, the common pattern is of Cyth1 expression and little or no Dnah17 expression. This indicates that BALB/cJ has the typical expression profile for these two genes, and that BALB/cByJ is atypical in the expression of both genes. BALB/cByJ mice are fully viable and appear normal, indicating that absence of Cyth1 expression and ectopic expression of Dnah17 has no major or visibly obvious consequence.

**Figure 2:**
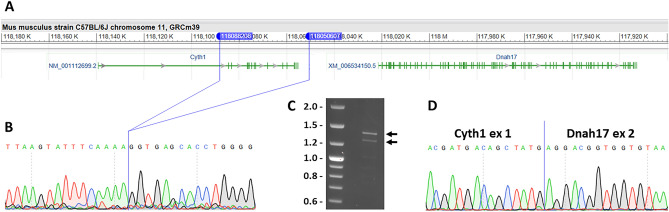
A 37.6 kb deletion in the Cyth1 gene of BALB/cByJ mice. (**A**) Map of the Cyth1-Dnah17 locus generated by the NCBI Sequence Viewer tool, edited to remove extraneous detail. The coordinates (NC_000077.7; GRCm39) of the deletion endpoints are indicated by the two blue markers. (**B**) Sequence of the genomic junction site of the deletion. (**C**) Amplification products from cDNA from BALB/cByJ heart using a forward primer from exon 1 of the Cyth1 gene and a reverse primer from exon 9 of the Dnah17 gene. Arrows point to the two major products; size markers in kb. (**D**) Sequence of the cDNA junction between Cyth1 exon 1 and Dnah17 exon 2 in the larger of the two products shown in panel (**C**); the smaller product has this same splice junction plus a downstream deletion of exon 7 of the Dnah17 gene.

In the mouse genome, Cyth1 and Dnah17 are immediately adjacent to each other on chromosome 11 (Fig. 2A). We evaluated BALB/cJ x cByJ F1 mice, which are heterozygous for all parental autosomal alleles, and observed that Cyth1 and Dnah17 transcripts were both expressed in F1 mice (Fig. 1B). In the full genome sequence of 53 inbred mouse strains from the Sanger Mouse Genomes Project, a closely linked SNP (rs261081071) in an intron of the Rbfox3 gene (400 kb from the Cyth1 transcriptional start site) is variant in BALB/cJ but not in any other sequenced mouse strain except the ancestrally distant CAST/EiJ. The BALB/cByJ genome has not yet been sequenced, but we determined that BALB/cByJ mice carry the reference allele at this SNP. By selecting for heterozygosity of this polymorphism, we backcrossed the Cyth1/Dnah17 locus from BALB/cByJ for 5 more generations into the BALB/cJ background. As in F1 mice, in heterozygous mice at N6 both genes were coexpressed (Fig. 1B). This indicates that the BALB/cJ and BALB/cByJ alleles behave independently and in a manner identical to their parental strain origins, and discounts the possibility of a mutation in a trans-acting factor affecting the expression of either gene from either strain source.

We addressed whether the reciprocal expression of Cyth1 and Dnah17 is a coordinated (interdependent) process. To do so, we obtained an engineered Cyth1 mutant allele on a C57BL/6J background^12^. This allele deletes exons 4–7 of the gene and does not encode a protein product, although a stable transcript is produced that is detectable with amplification primers downstream of the deletion. As with BALB/cByJ mice, Cyth1 null mice on a C57BL/6 J background are fully viable and appear normal. In these mice we observed no upregulation of Dnah17 (Fig. 1C). This indicates that the ectopic expression of Dnah17 in BALB/cByJ mice is not because of absence of Cyth1 expression, at least at the protein level. It is improbable that ectopic expression of Dnah17 suppresses Cyth1 expression as both genes are coexpressed in testes (per the ENCODE database) and in the hearts of BALB/cJ x BALB/cByJ F1 mice (Fig. 1B).

These observations are consistent with one or more mutational events having occurred within the Cyth1/Dnah17 locus in BALB/cByJ that accounts for the reciprocal expression of the two genes. From the RNA-Seq analysis, expression of all other genes in the vicinity of Cyth1 and Dnah17 was normal (Suppl. Table S2), also consistent with a single mutational event in BALB/cByJ that eliminated Cyth1 expression and caused expression of Dnah17.

### Molecular explanation for Cyth1 and Dnah17 expression

One possible explanation for the altered expression of both Cyth1 and Dnah17 in BALB/cByJ mice was an insertion or deletion. To address this, we amplified genomic DNA from BALB/cJ and BALB/cByJ mice using primers from various exons of the two genes. All primer pairs from the Dnah17 gene amplified successfully in the two strains. In contrast, primers from the 3’ end of the Cyth1 gene failed to amplify BALB/cByJ genomic DNA, although primers at the promoter and first exon of the gene amplified in both strains. We designed primers to progressively define regions of the Cyth1 gene that were or were not amplified from BALB/cByJ DNA, and ultimately identified a 37.6 kb deletion in the locus, between positions 118,050,607–118,088,208 (NC_000077.7, GRCm39 coordinates), from within the first intron of the Cyth1 gene to 4.4 kb 3′ of the last Cyth1 exon (Fig. 2A,B). The deletion breakpoints are 51.1 kb downstream of the Cyth1 transcription initiation site and 29.0 kb upstream of the first exon of the Dnah17 gene. There are no repetitive elements at either end of this deletion, indicating that it did not occur by recombination between homologous sequences.

The Cyth1 and Dnah17 genes are in the same transcriptional orientation. The genomic deletion in BALB/cByJ eliminates almost all of the coding sequence of the Cyth1 gene, but in addition brings the Cyth1 promoter and first exon and intron into much closer proximity to the Dnah17 gene. Furthermore, the deletion eliminates the transcriptional termination sequence at the 3’ end of the Cyth1 gene. Thus, a plausible explanation for Dnah17 expression in BALB/cByJ was a transcriptional start from the intact Cyth1 promoter, with readthrough transcription into and through the Dnah17 gene. Indeed, amplification of cDNA prepared from BALB/cByJ heart tissue using a forward primer from exon 1 of the Cyth1 gene and a reverse primer from exon 9 of the Dnah17 gene revealed two prominent products (Fig. 2C). Sequencing revealed that both correspond to splicing from the Cyth1 first exon to the Dnah17 second exon (Fig. 2D); the smaller product also excludes exon 7 (126nt long, exactly 42 amino acids) of the Dnah17 gene, which is a presumed natural alternative splice isoform of Dnah17 unrelated to Cyth1.

In the normal genes found in other mouse strains, Cyth1 mRNA translation begins in the first exon whereas Dnah17 translation begins in the second exon. In the Cyth1-Dnah17 fusion transcript, the two are out of frame with respect to each other. The Cyth1 translation start site is a perfect Kozak sequence (ACCATGG) and would be predicted to give rise to a 62 amino acid product from the fusion transcript if translated, containing the first 7 amino acids of Cyth1 followed by 55 ectopic codons before reaching a stop codon. In the fusion transcript there are two additional ATG sequences between the ATG codon of Cyth1 and the ATG codon of Dnah17. Thus, it is unlikely that this fusion transcript is translated in a manner that produces Dnah17 protein (although we cannot rule out the possibility that translation occurs at the Dnah17 ATG codon at some level). If so, the deletion in the Cyth1-Dnah17 locus in BALB/cByJ mice creates a full loss-of-function mutation of the Cyth1 gene and furthermore is unlikely to have any gain-of-function activity related to transcription (without translation) of the Dnah17 gene. The Dnah17 gene is highly expressed in testes and is required for sperm flagellar function; loss-of-function mutations of the Dnah17 gene cause infertility in mice, rats and humans^13,14^. Because BALB/cByJ male mice are fertile, the deletion in the Cyth1-Dnah17 locus in BALB/cByJ mice is unlikely to impact transcriptional regulatory elements that support Dnah17 testes gene expression.

### Cyth1 does not regulate cardiomyocyte ploidy

Our primary motivation in evaluating the Cyth1 locus was to consider if this gene contributed to differences in CM ploidy between BALB/cJ and BALB/cByJ mice. Relative to BALB/cJ and all other mouse strains, Cyth1 in BALB/cByJ is not expressed and is therefore a loss-of-function allele. If this variant allele has any phenotypic consequence it should behave in a recessive manner. As noted above, the autosomal influence on CM ploidy is recessive in BALB/cByJ relative to BALB/cJ.

**Figure 3:**
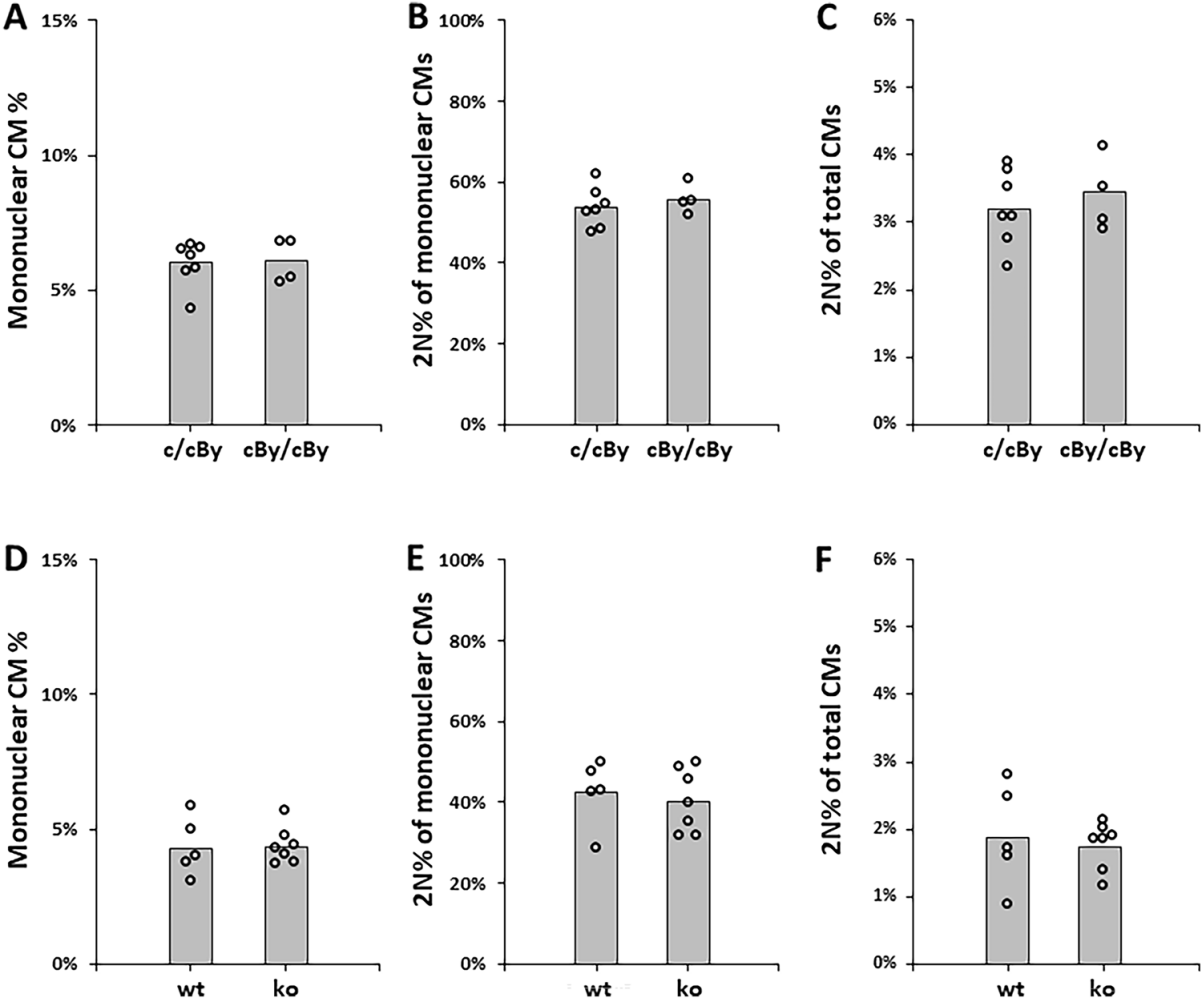
Cyth1 does not influence CM ploidy. (**A**) Mononuclear CM percentage in littermate adult mice (2 litters, males and females combined) that are either heterozygous or homozygous for the natural BALB/cByJ Cyth1 allele on an otherwise BALB/cJ-BALB/cByJ F1 genetic background. (**B**) The 2N (diploid) percentage specifically of the mononuclear CMs of the same mice as in panel (**A**). (**C**) The total diploid percentage of the same mice as in panels (**A**) and (**B**). (**D**) Mononuclear CM percentage in littermate adult mice (2 litters, males and females combined) that are either wild-type (wt) or homozygous for the engineered Cyth1 mutant allele (ko) on a fully C57BL/6 J genetic background. (**E**) The 2N (diploid) percentage of the mononuclear CMs of the same mice as in panel (**D**). (**F**) The total diploid percentage of the same mice as in panels (**D**) and (**E**).

We isolated ventricular CMs from adult mice to address whether CM ploidy is impacted by altered expression of Cyth1. For this analysis, we used mice in which the Cyth1-Dnah17 locus from BALB/cByJ was backcrossed into BALB/cJ for 7 generations, and then crossed heterozygous female mice to wild-type BALB/cByJ male partners. The offspring of these matings are therefore either heterozygous (cJ/cByJ) or homozygous (cByJ/cByJ) for the variant Cyth1-Dnah17 locus and are heterozygous for most other autosomal alleles across the genome except those closely linked to Cyth1. Male and female offspring were both used in the analysis; the design of cross ensures that all analyzed mice inherit at least one fully intact X chromosome from BALB/cJ (as noted above, the X-linked allele that influences CM ploidy is dominant in BALB/cJ and behaves in a CM-nonautonomous manner). As in our prior studies using the same method of analysis^6,8^, we first spread cells from a single cell preparation on slides and counted the percentage of CMs that were mononuclear or multinucleated. Then in a second step, mononuclear CMs were photographed in the blue channel and fluorescence signal quantified to determine nuclear ploidy (Suppl. Fig. S2A). We measured an equivalent mononuclear CM percentage and an equivalent nuclear ploidy within the mononuclear CM subpopulation regardless of Cyth1 genotype (Fig. 3A,B). Consequently, the diploid CM level was identical (Fig. 3C). In an analogous manner we evaluated CMs isolated from fully C57BL/6J-background mice that were either homozygous for the engineered Cyth1 null allele or wild-type (Fig. 3D-F). As in the BALB background, absence of Cyth1 in the C57BL/6 background did not impact overall CM ploidy or polyploid subtypes. CM total cell number was also not altered by Cyth1 genotype (Suppl. Fig. S2B). Although we did not directly measure neonatal CM cell cycle activity, it is unlikely that Cyth1 impacts this parameter if total cell number and ploidy subtypes are unchanged.

## Discussion

A new advance from this study was the identification and characterization of a natural null allele of Cyth1 in BALB/cByJ mice. Cytohesins are guanine nucleotide exchange factors (GEFs) that promote the activity of at least several members of the ADP ribosylation factor (ARF) and ARF-like (ARL) families of GTPases^15,16^. Arfs and Arls participate in a wide range of cellular functions, including karyokinesis and cytokinesis. Cyth1 full knockout is viable and has no externally visibly obvious phenotype^12^, but differences in CM ploidy would not be obvious without specific examination. Thus, Cyth1 was a plausible candidate to contribute to differences in CM ploidy between BALB/cJ and BALB/cByJ mice. The expectation in the crosses we made was that homozygosity of the relevant autosomal gene from BALB/cByJ would result in a lower frequency of diploid nuclei in mononuclear CMs. We did not see this in the BALB background, nor in the C57BL/6 background with an engineered null allele. We conclude that Cyth1 does not influence CM polyploidy.

One limitation of our analysis of CM ploidy is that we addressed the role of Cyth1 individually. It is possible that Cyth1 is additive in effect or redundant with other genes that are also polymorphic between BALB/cJ and BALB/cByJ. To experimentally test this, it would be informative to cross in the opposite direction as what was done here, i.e., to introduce the functional Cyth1 gene from BALB/cJ into the BALB/cByJ background, which we have not yet attempted. From RNA expression no other cytohesin gene is differentially expressed between the two strains, but we cannot exclude the possibility of a nonsynonymous point mutation in BALB/cByJ in one of these other cytohesin genes or in any other gene that might functionally interact with Cyth1 to impact CM ploidy. Because the BALB/cByJ genome has not yet been sequenced and made publicly available, such variants await discovery, as was the case for the large Cyth1 gene deletion identified in this report.

If Cyth1 is not involved in CM ploidy, as our data suggest, then other variants that distinguish BALB/cJ from BALB/cByJ must exist that are relevant to this phenotype. As described above, based on our prior F1 analysis the relevant genes are dominant in BALB/cJ. From the RNA-Seq analysis (Suppl. Table S1), other than Cyth1 and Dnah17 only a modest number of genes are differentially expressed. If an expression difference underlies phenotype difference it is more likely that the relevant gene is more highly expressed in BALB/cJ in order to be dominant. Almost nothing is known of Zfp433, the gene after Cyth1 that is most differentially expressed in BALB/cJ vs. BALB/cByJ heart. One complication in considering Zfp433 or other genes on this list is the consequence of Zhx2 mutation in BALB/cJ mice. Zhx2 functions as a transcriptional repressor^17^, so some genes might be more highly expressed in BALB/cJ hearts because of absence of Zhx2 in trans, rather than a de novo mutation in BALB/cByJ mice that reduces expression in cis. It might be possible to narrow the list of differentially expressed genes by further evaluating their expression in F1 mice, where Zhx2-dependent and Zhx2-independent expression differences would become clear. Two other possible scenarios to explain CM ploidy differences between the two BALB substrains should be noted. First, we limited the list of differentially expressed genes to greater than twofold, but a gene that has only a mild expression difference still could be phenotypically relevant. Alternatively, the important alleles that distinguish the two strains might be nonsynonymous coding variants that have functional differences without expression differences.

In Cyth1 engineered knockout mice, there is a modest deficiency in peripheral axon myelination, which is thought to relate to Cyth1 activation of Arf6 in Schwann cells^12^. We did not attempt to detect this phenotype in BALB/cByJ mice as it requires advanced techniques. For investigators interested in this feature or in other potential roles of cytohesins in vivo, if the engineered null allele is not accessible, the open availability of BALB/cByJ mice with a natural Cyth1 null allele means that such studies can be readily conducted.

## Methods

Review of experimental procedures. All studies were conducted under the oversight of the Medical University of South Carolina (MUSC) Office of Research Compliance, which reports to the MUSC Vice President for Research. Studies involving biosafety matters were reviewed and approved by the MUSC Institutional Biosafety Committee (protocol 2023-01604). Mouse studies were conducted under the oversight of the MUSC Institutional Animal Care and Use Committee (protocol 2018-00642), and all experiments were performed in accordance with relevant guidelines and regulations, including ARRIVE guidelines.

Mice. BALB/cJ, BALB/cByJ and C57BL/6J mice were obtained from the Jackson Laboratory or were bred in-house using mice originating from the Jackson Laboratory. Sperm from Cyth1 heterozygous mice was obtained from the JCRB Laboratory Animal Resource Bank, National Institutes of Biomedical Innovation, Health and Nutrition (NIBIOHN; strain nbio 247) and the knockout allele rederived by fertilization of wild-type C57BL/6J eggs by the MUSC Transgenic Core.

RNA-Seq. Litter sizes from matings of BALB/cJ or BALB/cByJ mice were controlled to 4–6 pups per female to reduce variation due to nursing conditions. Ventricular tissue from 3–4 P4 mice (P0 = day of observation of newborn pups) per sample was dissected and snap frozen in ethanol cooled with dry ice. RNA was extracted in Trizol (Invitrogen) with homogenization, purification was then performed using the RNeasy kit (Qiagen). 4 separate samples of RNA from BALB/cJ and 3 from BALB/cByJ passed RNA quality assessment using a BioAnalyzer, and these were sent to BGI Genomics for library preparation and sequencing and for evaluation of differentially expressed genes. Results were filtered for significance (q < 0.05).

RT-PCR. Tissue was washed in 1 × PBS then homogenized on ice in Trizol. Following chloroform extraction and isopropanol precipitation, RNA was dissolved in DEPC-treated water. cDNA was prepared using the SuperScript III First-Strand Synthesis System (Invitrogen 18080-051). PCR utilized the following gene specific primers:

Cyth1 Forward Primer: ACAAGCCTACGGTGGAGAGA (exon 8)

Cyth1 Reverse Primer: CGGATACTCAGGTTCTCCAGG (exon 11)

Dnah17 Forward Primer: TCCTACGTGTACGGGCTCTT (exon 80)

Dnah17 Reverse Primer: GCATCAGACGGGCACAATG (exon 81)

Gapdh Forward Primer: GGAAGGGCTCATGACCACA

Gapdh Reverse Primer: CCCTGTTGCTGTAGCCGTA

For amplification of the Cyth1-Dnah17 fusion transcript, PCR utilized the following primers:

Cyth1 Forward primer: TGTGGCGAGCTGGTGTCTGGCA (exon 1)

Dnah17 Reverse primer: ATGGTCTGCACGCGGTGAAAGAAG (exon 9)

Rbfox3 SNP for locus genotyping. The SNP rs261081071 in the Rbfox3 gene was initially identified in a search using the SNP database tool at phenome.jax.org/genotypes, and then confirmed to be polymorphic between BALB/cJ and BALB/cByJ by amplification of the flanking genomic DNA and direct sequencing. To use this SNP as a marker of the closely linked Cyth1-Dnah17 locus in backcrosses from BALB/cByJ into BALB/cJ, double mismatch allele-specific quantitative PCR^18^ was used for genotyping. For this, the common reverse primer TCGCAGCCTACCTTCAACC was paired with the allele-specific forward primers TCTTATGTGTATCCAGAGACCA (BALB/cJ) or TCTTATGTGTATCCAGAGACCG (BALB/cByJ), with amplification conducted on a BioRad CFX96 real-time thermocycler using SYBR Green Supermix (Bio-Rad, 1725271).

Ventricular single cell preparations. Hearts were removed from anesthetized (by isoflurane vapor inhalation) heparin-treated mice (Sigma-Aldrich H3393-100KU, 2 mg per mouse in 0.1 ml PBS) and quickly attached to a Langendorff perfusion rig with a pump rate of 3 ml/min. Hearts were perfused with 9 ml calcium-free Tyrode’s solution, then with 20 ml collagenase type II (1 mg/ml; Gibco 17101015) dissolved in calcium-free Tyrode’s solution. Subsequently, hearts were lightly fixed by perfusion of 5 ml 2% paraformaldehyde in PBS, then the lower 2/3 of ventricular tissue (from apex to near the base but with care to avoid valves) was separated with forceps into Kruftbrühe solution and gently triturated using a transfer pipet to prepare a single cell suspension. This was filtered by gravity through a 300μ nylon mesh, then fixed by adding an equal volume of 4% PFA in PBS at room temperature with rocking for 5 min, and fixation terminated by adding an equal volume of 5% BSA in PBS. After centrifugation (1000 rpm, 1 min) and removal of the supernatant, cells were suspended in PBS and stored at 4 °C.

Ploidy evaluation. Aliquots of heart cells were stained for 15 min with Hoechst 33342 (ThermoFisher Scientific, 1:4000) at room temperature, then washed 3 times with PBS. A small volume of the final cell suspension was combined with an equal volume of ProLong Gold Antifade mountant (Thermo Fisher Scientific, P36930) to prepare microscope slides. Mononuclear CM percentage was determined by counting a minimum of 600 cells per preparation using autofluorescence to visualize CMs. Nuclear ploidy of mononuclear CMs was measured by fluorescence signal intensity in digital photographs (quantified with ImageJ) normalized to the median fluorescence intensity of individual nuclei of binucleated CMs in the same images.

### Supplementary Information

Supplementary Information.

## Data Availability

All data generated or analysed during this study are included in this published article (and its Supplementary Information files). RNA-Seq data have been deposited in NCBI as GSE268101.
