# Supplementary material for: A natural loss-of-function deletion of the cytohesin 1 (Cyth1) gene in BALB/cByJ mice does not impact cardiomyocyte polyploidy
Source: Sci Rep. 2024 Jun 10;14:13333. doi: 10.1038/s41598-024-63667-8 (PMC11164939; doi:10.1038/s41598-024-63667-8)
Supplement: Supplementary file 1 — Supplementary Information. [file 41598_2024_63667_MOESM1_ESM.pdf]

Supplementary Information

for Song et al.

A natural loss-of-function deletion of the cytohesin 1 (Cyth1) gene in BALB/cByJ mice does not impact cardiomyocyte polyploidy

2 Supplementary Tables
2 Supplementary Figures

Higher in BALB/cJ

gene_id	gene_symbol	FC
19157	Cyth1	184.4
73610	Zfp433	17.9
12504	Cd4	4.5
331188	Zfp781	4.4
330096	Shisa3	4.0
227929	Cytip	3.7
107566	Arl2bp	3.4
66898	Baiap2l1	3.3
71886	2310002L09Rik	3.0
20743	Sptbn2	2.9
13116	Cyp46a1	2.6
13489	Drd2	2.5
21367	Cntn2	2.4
244867	Arhgap20	2.3
66404	Rtf2	2.3
108167806	Gm46221	2.3
12797	Cnn1	2.2
211323	Nrg1	2.2
268857	Nlrc3	2.2
244810	AW551984	2.1
108168067	Gm46403	2.0

Higher in BALB/cByJ

gene_id	gene_symbol	FC
100041621	Gm3435	85.5
69926	Dnah17	54.9
387609	Zhx2	18.0
54612	Sfrp5	4.3
21955	Tnnt1	3.4
213603	Slc44a3	3.4
17318	Mid1	3.3
74558	Gvin1	3.3
19049	Ppp1r1b	3.2
76933	Ifi27l2a	3.1
109801	Glo1	3.1
15957	Ifit1	3.0
665155	Srp54b	3.0
333182	Cox6b2	3.0
100041546	Ly6c2	2.9
15019	H2-Q8	2.8
81904	Cacng7	2.8
54378	Cacng6	2.5
24110	Usp18	2.5
105243	Slc9a3	2.5
320204	Etfbkmt	2.5
11831	Aqp6	2.4
626578	Gbp10	2.4
108167855	Gm46255	2.4
14695	Gnb3	2.3
23962	Oasl2	2.3
54123	Irf7	2.3
77462	Tmem116	2.3
100702	Gbp6	2.3
59011	Myoz1	2.3
18054	Ngp	2.2

Supplementary Table S1. Differentially expressed genes identified by RNA-Seq analysis between BALB/cJ and BALB/cByJ neonatal hearts; FC=fold change. Genes with differences in expression >2-fold and surpassing a $q<0.05$ statistical value are shown.

Annotated genes near Cyth1/Dnah17

gene_id	gene_symbol	chr:pos (GRCm39)	FC (c/cBy)
74136	Sec14l1	11:117005994	-1.0
53860	Sept9	11:117090487	-1.1
217351	Tnrc6c	11:117545115	1.1
217353	Tmc6	11:117656811	1.1
217356	Tmc8	11:117672902	1.1
77727	6030468B19Rik	11:117688486	-2.4
20973	Syngr2	11:117700494	1.1
21877	Tk1	11:117706352	1.1
71562	Afmid	11:117716750	1.0
11799	Birc5	11:117740077	1.2
546519	Tmem235	11:117751578	4.9
71776	Tha1	11:117758778	1.0
12702	Socs3	11:117856905	-1.1
74451	Pgs1	11:117877118	1.2
69926	Dnah17	11:117912549	-55.0
19157	Cyth1	11:118054996	184.4
72344	Usp36	11:118150477	1.5
21858	Timp2	11:118191887	1.2
58251	Cep295nl	11:118223186	1.5
19039	Lgals3bp	11:118283573	-1.6
76025	Cant1	11:118297115	1.2
56745	C1qtnf1	11:118319029	-1.1
217364	Engase	11:118367655	-1.2
52897	Rbfox3	11:118380588	1.1
12416	Cbx2	11:118913845	1.2
30951	Cbx8	11:118929262	1.3
12418	Cbx4	11:118968399	-1.1

Supplementary Table S2. Expression of annotated genes within 1Mb of Cyth1 and Dnah17, showing measured fold change (FC) by RNA-Seq analysis in BALB/cJ compared to BALB/cByJ. Other than Dnah17 and Cyth1 (boxed, in center), no other differences were statistically significant at a threshold of $q<0.05$.

Suppl. Fig. S1. Original images of gels cropped in Fig. 1

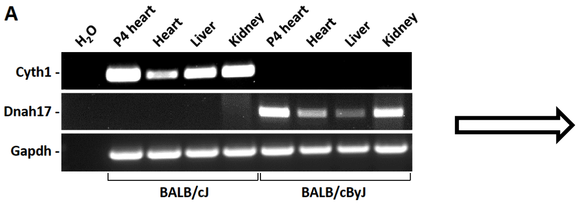

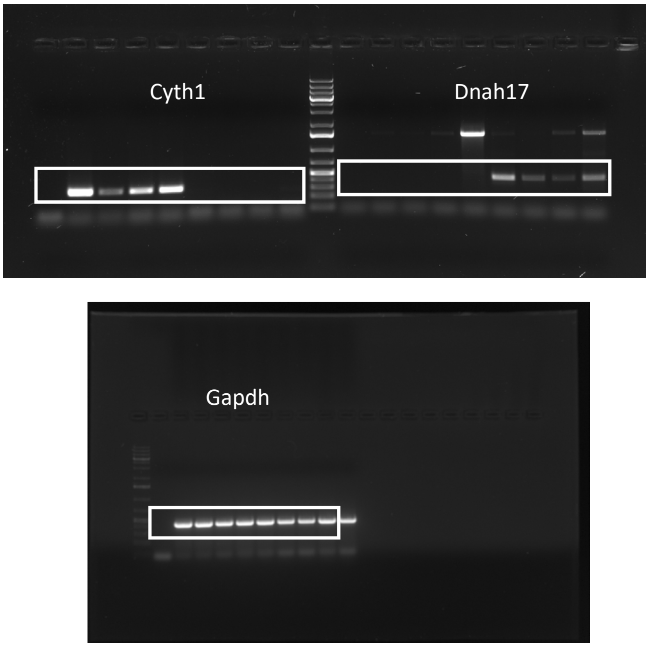

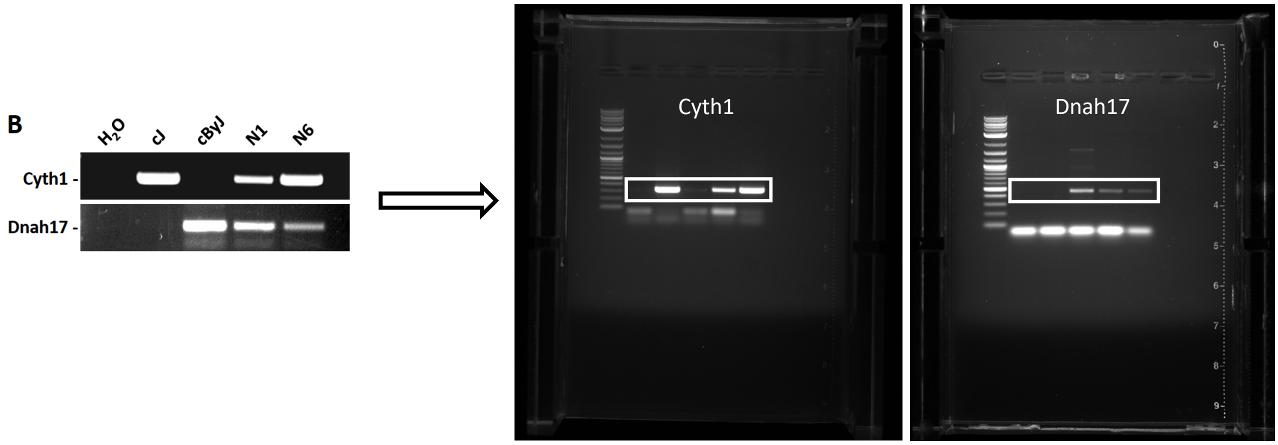

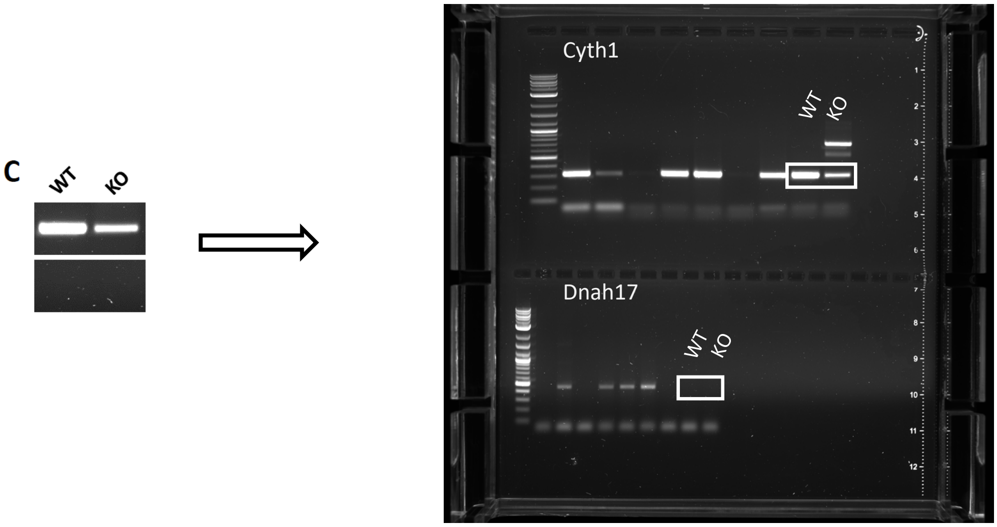

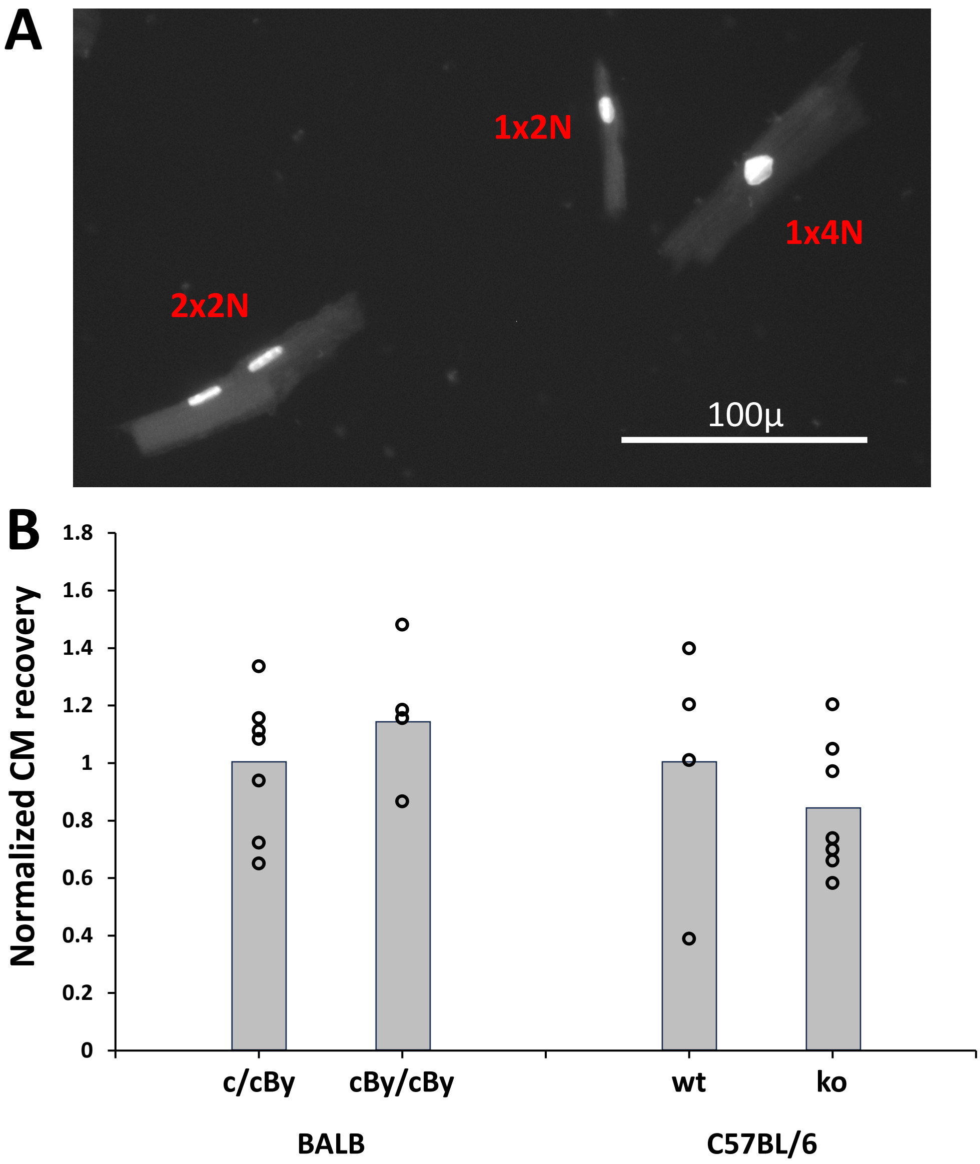

Supplementary Fig. S2. Ventricular single cell suspensions for ploidy analysis. **A.** One illustrative photograph of isolated adult ventricular CMs with the ploidy subtype of individual cells noted. **B.** Normalized total number of recovered CMs from the same cell preparations used for analysis in Fig. 3. Because only a portion of each ventricle was used to derive cell suspensions (see Methods), each data point is an estimate based on the assumption that a roughly similar amount of ventricle tissue was used in each. The data are normalized to the level for each strain that expresses a function Cyth1 gene. From estimated yields, a normalized number of 1 corresponds to an absolute number of 1.2E6 CMs per ventricle for both BALB c/cBy and C57BL/6 wt mice. One sample of C57BL/6 wild-type cells that was included in Fig. 3 was no longer available when this analysis was performed.
